# Insecticidal and Nematicidal Activities of Novel Mimosine Derivatives 

**DOI:** 10.3390/molecules200916741

**Published:** 2015-09-14

**Authors:** Binh Cao Quan Nguyen, Jamnian Chompoo, Shinkichi Tawata

**Affiliations:** 1 Department of Bioscience and Biotechnology, The United Graduate School of Agricultural Sciences, Kagoshima University, Korimoto 1-21-24, Kagoshima 890-8580, Japan; E-Mail: ncqbinh@gmail.com; 2 Department of Agronomy, Faculty of Agriculture, Kasetsart University, Kamphaeng Saen Campus, Nakhon Pathom 73140, Thailand; E-Mail: mcmine56@yahoo.com; 3 Department of Bioscience and Biotechnology, Faculty of Agriculture, University of the Ryukyus, Senbaru 1, Nishihara-Cho, Okinawa 903-0213, Japan

**Keywords:** mimosine, mimosinol, insecticidal, nematicidal, tyrosinase, acetylcholinesterase

## Abstract

Mimosine, a non-protein amino acid, is found in several tropical and subtropical plants, which has high value for medicine and agricultural chemicals. Here, in continuation of works aimed to development of natural product-based pesticidal agents, we present the first significant findings for insecticidal and nematicidal activities of novel mimosine derivatives. Interestingly, mimosinol and deuterated mimosinol (d-mimosinol) from mimosine had strong insecticidal activity which could be a result of tyrosinase inhibition (IC_50_ = 31.4 and 46.1 μM, respectively). Of synthesized phosphoramidothionate derivatives from two these amino alcohols, two compounds (**1a** and **1b**) showed high insecticidal activity (LD_50_ = 0.5 and 0.7 μg/insect, respectively) with 50%–60% mortality at 50 μg/mL which may be attributed to acetylcholinesterase inhibition. Compounds **1a** and **1b** also had strong nematicidal activity with IC_50_ = 31.8 and 50.2 μM, respectively. Our results suggest that the length of the alkyl chain and the functional group at the C_5_-position of phosphoramidothionates derived from mimosinol and d-mimosinol are essential for the insecticidal and nematicidal activities. These results reveal an unexplored scaffold as new insecticide and nematicide.

## 1. Introduction

Organophosphorus compounds are a highly diverse class of organic chemicals with many uses [1], and form one of the most important groups of modern pesticides [2]. The advantageous properties of phosphorus compounds are a result of their relatively low stability, their decomposition to products that are not toxic to humans and animals, and their low rate of use per unit treated area [3]. Another important feature of these compounds is that their action is highly selective [4]. Designed compounds containing phosphorus may be less dangerous in use without losing their values as effective pesticides [3]. Utilization of organophosphorus pesticides is favored over organochlorine compounds because of their ability to degrade more readily in the environment [5]. However, overuse of these compounds can lead to the development of resistance in pests [6]. Pest resistance has led to the need for development of new non-persistent and non-toxic pesticides with selective activity to help maintain stable food production.

Acetylcholinesterase (AChE) functions in termination of the neurotransmission by breaking down the neurotransmitter acetylcholine at cholinergic synapses in all animals. In insects, it serves as a major target for anti-cholinesterase insecticides, and constitutes a common mechanism of insecticide resistance through its reduced sensitivity to the insecticides [7]. Tyrosinase plays important roles in normal developmental processes in insects; inactivation of tyrosinase activity can lead to the loss of insect defense mechanisms or to abnormal body softening which could lead to pest control [8]. Thus, these two enzymes serve as targets for the development of novel anti-cholinesterase insecticides and bioinsecticides [7,8].

Mimosine [β-[*N*-(3-hydroxy-4-oxypyridyl)]-α-aminopropionic acid] is a non-protein amino acid containing an alanine side chain bound to the nitrogen atom of a hydroxypyridone ring. It is found in several tropical and subtropical plants, which possesses a wide range of biological activities and has strong medicinal properties [9] including anti-viral [10], anti-inflammation [11], and anti-cancer effects [12]. Mimosine is also responsible for herbicidal activity [13], and can suppress insect growth along with its effects on the activity of trehalase, invertase, and amylase in a dose-dependent manner [14]. However, a review of the literature reveals mimosine derivatives with insecticidal and nematicidal activities have not been thoroughly reported yet.

Cyclic phosphorus compounds such as salithion, 4-isobutyl-2-methoxy-1,3,2-oxazaphospholidine 2-sulfide and 2-methoxy-5-phenyl-1,3,2-oxazaphospholidine 2-sulfide have been shown in connection with strong insecticidal activity [15]. The insecticidal activity has also been shown in five-membered cyclic phosphoramidates and phosphoramidothiolates [16]. Moreover, Eto *et al*. [17] have reported that five-membered cyclic phosphoramidothionates from amino alcohol of l-leucine and other amino acids have insecticidal activity by inhibiting acetylcholinesterase. Taken together, in this study, using mimosine (non-protein amino acid) as starting material, we identified insecticidal activity in amino alcohols derived from mimosine, and we synthesized a family of five-membered cyclic phosphoramidothionate derivatives from these alcohols to assess their insecticidal and nematicidal activities. Their insecticidal mode of action was also tested on acetylcholinesterase and tyrosinase inhibition. To our knowledge, this is the first report on the synthesis of novel amino alcohols and phosphoramidothionate derivatives from mimosine, and the investigation of their insecticidal and nematicidal activities.

## 2. Results and Discussion

### 2.1. Synthesis

**Figure 1:**
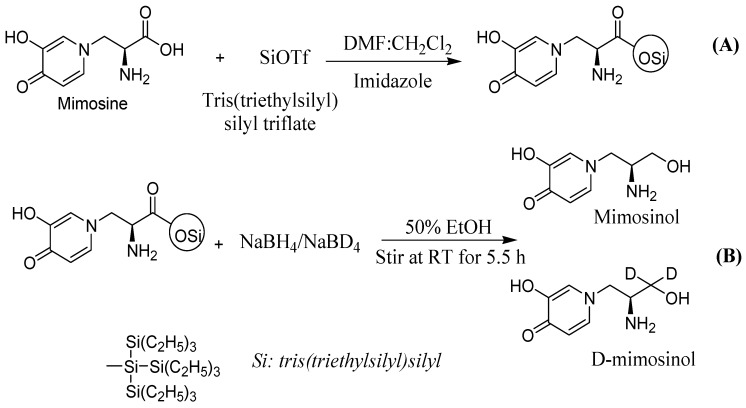
Scheme for reduction of mimosine into mimosinol and d-mimosinol. (**A**) Installation of tris(triethylsilyl)silyl group into mimosine; and (**B**) reduction of mimosine ester to mimosinol and d-mimosinol.

**Figure 2:**
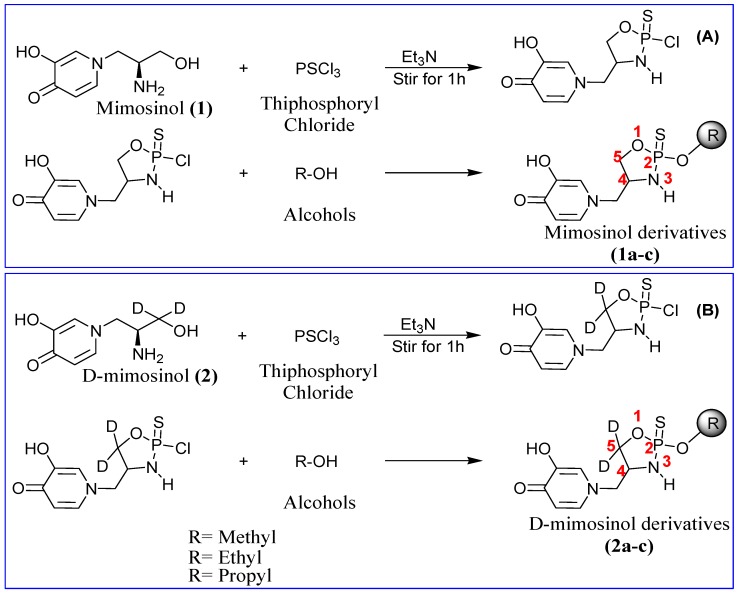
General route for synthesis of phosphoramidothionate derivatives. (**A**) Scheme for synthesis of derivatives from mimosinol; (**B**) Preparation of derivatives from deuterated mimosinol (d-mimosinol).

In this study, we describe a route for synthesis of amino alcohols and their phosphoramidothionates from mimosine. Mimosinol and d-mimosinol were prepared using a three-step procedure. First, tris(triethylsilyl)silyl triflate was generated *in situ* by mixing tris(triethylsilyl)silane and triflic acid. Second, installation of the supersilyl group (tris[triethylsilyl]silyl) was achieved by treatment of mimosine with tris(triethylsilyl)silyl triflate in the presence of imidazole and DMF:CH_2_Cl_2_ (1:1). Finally, mimosine supersilyl ester was reduced to mimosinol and d-mimosinol using sodium borohydride or sodium borodeuteride with yields of 95% and 73.5%, respectively (Figure 1). Phosphoramidothionate derivatives were produced by the reaction of mimosinol and d-mimosinol with thiophosphoryl chloride (Figure 2). The chloride atom of intermediates was displaced by bulky alkyl group under the triethylamine catalyst to afford compounds **1a**–**c** (28.9%–39.6% yield) from mimosinol and compounds **2a**–**c** (10%–15% yield) from d-mimosinol.

### 2.2. Insecticidal Activity

Insect mortality rate was evaluated by topical application and a no-choice contact method. Mimosinol and its derivatives (**1a**–**c**) had strong insecticidal activity with LD_50_ values in the ranges from 0.5 to 1.2 μg/insect, whereas d-mimosinol and its derivatives (**2a**–**c**) exhibited lower insecticidal activity (LD_50_ from 1.2 to 3.1 μg/insect) (Table 1). Among all tested compounds, compounds **1b** and **1a** showed promising activity against insects (52%–62% and 71%–88% mortality at 50 and 100 μg/mL after seven days treatment, respectively; Table 2), which is comparable to the commercial insecticide rotenone (Table 1 and Table 2).

**Table 1:** LD_50_ values of mimosine derivatives against termites by topical application.

Compound	LD_50_ (μg/insect) *^i^*
Mimosinol	1.2 ± 0.2 de
**1a**	0.5 ± 0.1 fg
**1b**	0.7 ± 0.1 ef
**1c**	0.8 ± 0.1 ef
d-mimosinol	1.8 ± 0.3 d
**2a**	1.2 ± 0.2 de
**2b**	2.1 ± 0.4 c
**2c**	3.1 ± 0.5 b
Mimosine	54.1 ± 5.9 a
Rotenone	0.8 ± 0.2 ef
Fenitrothion	0.025 ± 0.0 g

*^i^* Different letters in the same column indicate the existence of significant difference statistically. Data have statistical significance at *p* ≤ 0.01. The results are means ± SE of four replications for each treatment.

**Table 2:** Insecticidal activity of mimosine derivatives using a no-choice contact method.

Compound	Mortality (%)		
	250 μg/mL *^ii^*	100 μg/mL	50 μg/mL
Mimosinol	68.5 ± 0.6 c	52.5 ± 2.7 de	38.1 ± 1.3 cde
**1a**	90.6 ± 1.3 a	88.8 ± 0.9 a	62.5 ± 1.8 a
**1b**	84.4 ± 2.2 ab	71.9 ± 1.3 b	52.5 ± 3.5 ab
**1c**	77.5 ± 0.9 b	59.4 ± 1.3 cd	45.6 ± 0.4 bc
d-mimosinol	60.6 ± 0.6 cd	53.8 ± 3.8 de	33.1 ± 1.9 de
**2a**	82.5 ± 1.3 ab	70.6 ± 0.6 b	48.8 ± 2.5 bc
**2b**	63.8 ± 0.0 c	52.5 ± 2.5 de	42.5 ± 2.5 bcd
**2c**	52.5 ± 3.8 d	44.4 ± 1.9 e	29.4 ± 1.9 e
Mimosine	9.1 ± 2.0 e	7.5 ± 1.0 f	2.5 ± 1.1 f
Rotenone	80.0 ± 0.9 b	68.1 ± 1.3 bc	48.8 ± 0.9 bc
**Compound**	**Mortality (%)**		
	**15 μg/mL**	**10 μg/mL**	**5 μg/mL**
Fenitrothion	83.8 ± 1.9	69.4 ± 0.6	26.9 ± 1.3

*^ii^* Compound dose. Data have statistical significance at *p* ≤ 0.01. Different letters in the same column indicate the existence of significant difference statistically. The results are means ± SE of two independent experiments with four replications for each treatment.

### 2.3. Tyrosinase and AChE Inhibition

**Figure 3:**
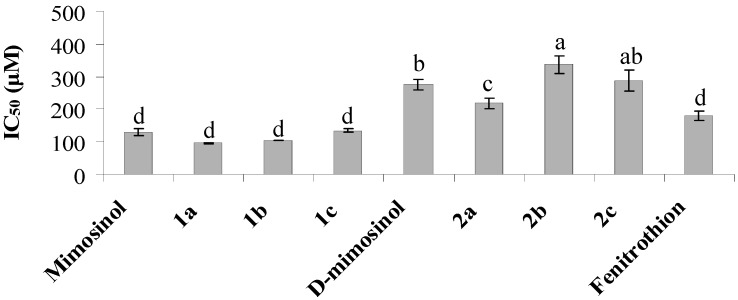
Inhibition of acetylcholinesterase (AChE) activity from termite whole bodies of mimosinol, deuterated mimosinol (d-mimosinol) and their derivatives (**1a**–**c**, **2a**–**c**). Values are means of two independent experiments ± SE. IC_50_ value represents 50% inhibition of AChE activity of tested compounds. The different letters above bars represent significant differences statistically at *p* ≤ 0.01. Mimosine and kojic acid were not active for AChE inhibition with IC_50_ of 1528 and 5477 μM, respectively.

**Figure 4:**
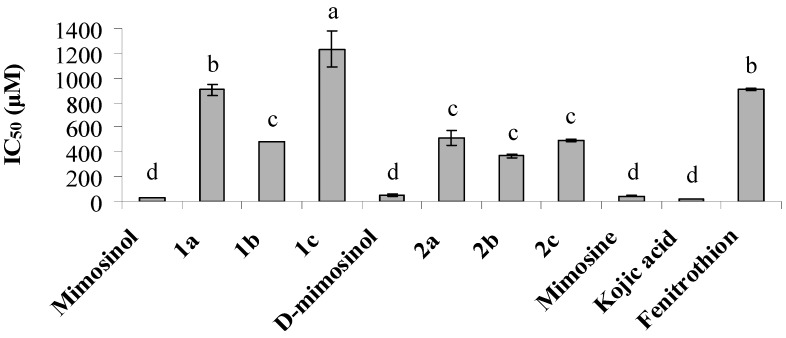
Inhibition of mimosinol, deuterated mimosinol, and their derivatives (**1a**–**c**, **2a**–**c**) against tyrosinase. Values are means of two independent experiments ± SE. IC_50_ value represents 50% inhibition. The different letters above bars represent significant differences statistically at *p* ≤ 0.01.

To determine insecticidal mode of action, AChE and tyrosinase inhibition was investigated. Mimosinol and its derivatives (**1a**–**c**) had strong AChE inhibitory activity that was significantly better than that of d-mimosinol and its derivatives (**2a**–**c**) (Figure 3). The IC_50_ values against AChE of compounds **1a** and **1b** were 95.9 and 104.0 μM, respectively. Fenitrothion showed AChE inhibitory activity, with IC_50_ of 181.5 μM. Interestingly, mimosinol and d-mimosinol showed strong inhibitory activity against tyrosinase, with IC_50_ values of 31.4 and 46.1 μM, respectively. The other synthesized derivatives displayed low tyrosinase inhibitory activity (Figure 4).

### 2.4. Nematicidal Activity of Mimosine Derivatives

**Figure 5:**
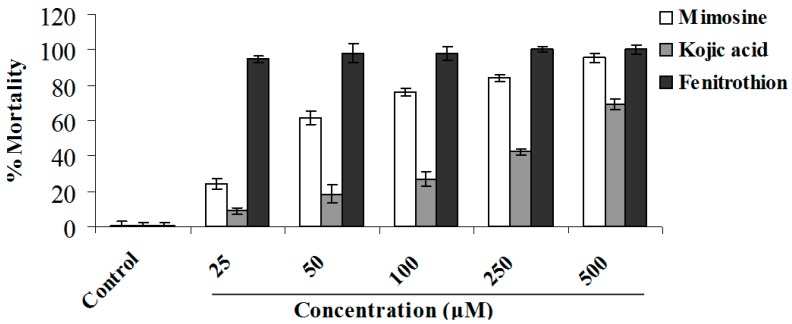
Nematicidal activity of mimosine against *Caenorhabditis elegans*. Dead and active nematodes were recorded after 48 h treatment. Nematode mortality rate was calculated as a percentage relative to that recorded in the control group. Values are means of two independent experiments ± SE. Mimosine and kojic acid had the IC_50_ values of 16.8 and 327.9 μM, respectively.

**Figure 6:**
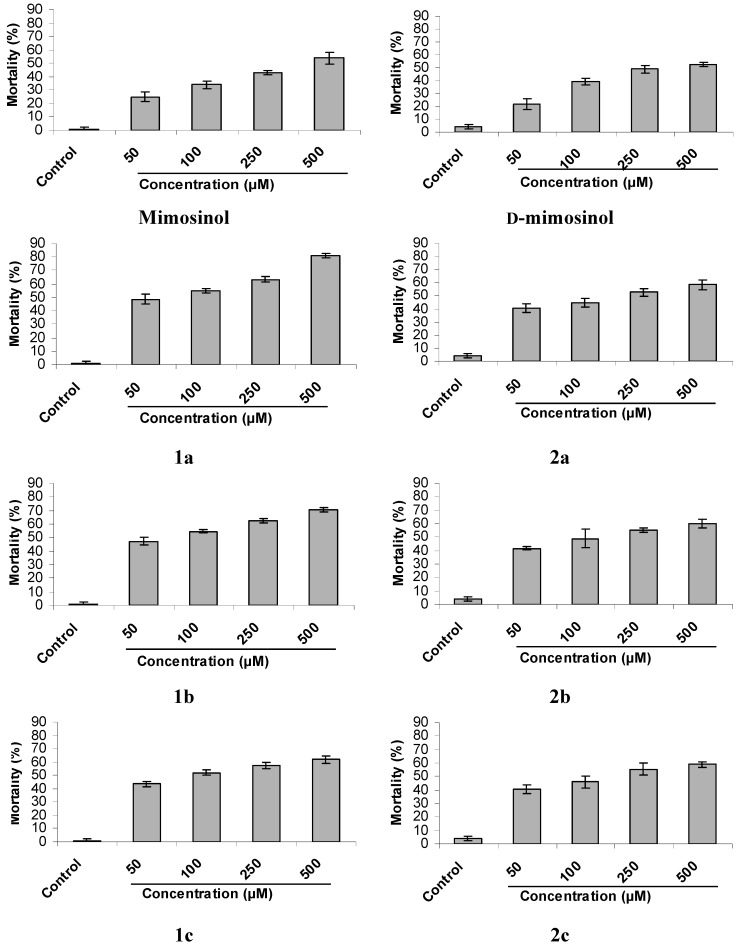
Nematicidal activity of mimosinol and its derivatives (**1a**–**1c**), deuterated mimosinol (d-mimosinol) and its derivatives (**2a**–**2c**) against *Caenorhabditis elegans*. Dead and active nematodes were evaluated after 48 h treatment. Nematode mortality rate was calculated as a percentage relative to that recorded in the control group. Values are means of two independent experiments ± SE. The IC_50_ values of mimosinol (376.2 μM), **1a** (31.8 μM), **1b** (50.2 μM), and **1c** (116.1 μM). The IC_50_ values of d-mimosinol (390.0 μM), **2a** (251.6 μM), **2b** (186.7 μM), and **2c** (218.1 μM).

The assay for nematicidal activity against *C. elegans* was conducted using the 96-well microplate method. Interestingly, mimosine had high nematicidal activity, with an IC_50_ value of 16.8 μM (Figure 5). Mimosinol and d-mimosinol showed moderate nematicidal activity, with IC_50_ values of 376.2 and 390.0 μM, respectively (Figure 5 and Figure 6). Nematicidal activity differed among functional groups. Phosphoamidothionate derivatives derived from mimosinol had activity better than those derived from d-mimosinol. Compounds **1a** and **1b** exhibited particularly strong nematicidal activity, with IC_50_ values of 31.8 μM and 50.2 μM, respectively. The other derivatives had pronounced nematicidal activity.

### 2.5. Discussion

The chemical modification of amino acid has been attempted for searching a new lead compound for insecticides. Since discovery of l-leucine amino acid as a neuroactive substance in the blood of DDT-treated silk-worm larvae, a new series of insecticidal five-membered cyclic phosphoramidothionates from this amino acid and other natural ones have been developed [17]. Therefore, in this study, we used mimosine, a natural non-protein amino acid, as starting material for developing new five-membered cyclic phosphoramidothionates for insecticidal and nematicidal activities. We chose termites as a representative insect because of their significance worldwide and particularly in Japan [18]. As shown in Table 1, mimosinol and d-mimosinol from mimosine had much higher insecticidal activity (>30 times) than mimosine, suggesting that the importance of modification of –COOH group of mimosine for increasing insecticidal activity. For the mimosinol- and d-mimosinol-derived phosphoramidothionates, the alkyl group in -OR affects insecticidal activity of these compounds. The increase of alkyl side chain will lead to the reduction of insecticidal activity (Table 1 and Table 2).

Development of insecticides requires the full understanding of molecular mechanism of the insecticides that we used into the target insects. Many organophosphate insecticides including five-membered cyclic phosphorous insecticide inhibit AChE, which results in the prolonged binding of acetylcholine to its postsynaptic receptor, leading to the death of target insects by a prolonged neuroexcitation [15,16,17,19]. Tyrosinase, a copper-containing enzyme, is also a potential candidate for novel bioinsecticide development [8]. Tyrosinase catalyzes the hydroxylation of monophenols and oxidation of *o*-diphenols to *o*-quinones which polymerise spontaneously to form dark macromolecular pigments, such as insoluble melanin. Moreover, *o*-quinones cross link with cuticular structural proteins and chitin, which result in hardening of the cuticle. Therefore, inhibition of tyrosinase could cause abnormal body softening [20]. Interestingly, we provide new findings that the insecticidal activity of mimosinol and d-mimosinol could be due to tyrosinase inhibition; therefore, mimosinol and d-mimosinol could inhibit the development and molting of insects. It is interesting to test whether mimosinol and d-mimosinol inhibit tyrosinase expression *in vivo* or not. Since the insect tyrosinase was labile and easy to inactivate during purification, its inactivate precursor, prophenoloxidase (PPO), was more stable [8]. Thus, we are planning to test whether these two compounds inhibit PPO expression in insects or not. On the other hand, in contrast, the insecticidal activity of phosphoramidothionate derivatives of mimosinol and d-mimosinol, including compounds **1a** and **1b**, could be attributed to AChE inhibition when compared to tyrosinase inhibition (Figure 3 and Figure 4). Figure 3 showed that AChE inhibitory activity was influenced by replacing two hydrogens at the C_5_-position. One plausible explanation is that the functional group at C_5_-position may contribute to the interaction with the insect nerve.

Nematodes attack a wide variety of organisms and present a major challenge to humans and agriculture [21]. The results showed that mimosine and compounds **1a** and **1b** are promising compounds for nematicide. The nematicidal activity was influenced by substituent of C_5_-position rather than the alkyl group (-OR) (Figure 2 and Figure 6).

In fact, the biological activities of cyclic phosphoramidates in general are greatly affected by the ring size, the exocyclic ester group and alkyl group at C_4_-position including the configuration there. The five-membered ring is necessitated for the biological activities [17]. Our investigation on structure-activity relationship has seen that other interesting factors affect considerably the activity of five-membered cyclic phosphoramidothionates. It was found that the length of the alkyl chain (-OR) was inversely proportional to insecticidal and nematicidal activities. Substitution of two hydrogens at the C_5_-position on five-membered ring would also lead to reduced insecticidal and nematicidal activities.

## 3. Experimental Section

### 3.1. Chemicals and Reagents

Tris(triethylsilyl)silane, rotenone, 5,5′-dithiobis-(2-nitrobenzoic acid), acetylthiocholine iodide, and sodium borodeuteride were obtained from Sigma-Aldrich (Tokyo, Japan). l-tyrosine and sodium borohydride were purchased from Wako Pure Chemical Industries (Osaka, Japan). Trifluoromethanesulfonic acid was obtained from Nacalai Tesque (Kyoto, Japan), and thiophosphoryl chloride was obtained from Tokyo Chemical Industry Co. (Tokyo, Japan). Fenitrothion was obtained from Dr. Ehrenstorfer GmbH (Augsburg, Germany). Mushroom tyrosinase was obtained from Sigma-Aldrich (Saint Louis, MO, USA), and kojic acid was purchased from Kanto Chemical Co. (Tokyo, Japan). Unless otherwise mentioned, all reagents were of analytical grade and were obtained from Wako Pure Chemical Industries and Kanto Chemical Co., Japan. The ^1^H- and ^13^C-NMR spectra were recorded on a JEOL JNM-ECA400 spectrometer (JEOL Resonance, Tokyo, Japan) in CDCl_3_. Chemical shifts were expressed in parts per million (δ) relative to tetramethylsilane (TMS).

### 3.2. Mimosine Isolation from Leucaena leucocephala Leaves

Samples of *Leucaena leucocephala* leaves were collected at the Faculty of Agriculture, University of the Ryukyus, Okinawa, Japan (lat. 26°N, long. 127°E). Fresh leaves (1.5 kg) were boiled in 5 L water for 10 min. The cooled liquid extract was sieved by suction filtration in a shaking bath (SB-20; As One, Osaka, Japan), and the filtrate was mixed with ion-exchange resin (2 kg) (Mitsubishi Chemical, Tokyo, Japan), stirred for 30 min, and left overnight. The resin was rinsed with distilled water 5–6 times, and 5 L of 80% ethanol was added dropwise to remove chlorophyll. Mimosine was dissolved from the resin with dropwise addition of 6 L of 2 N NH_4_OH. The liquid extract was concentrated to a final volume of 300 mL at 40 °C under reduced pressure. The solution was adjusted to pH 4.5–5.0 with 6 N HCl, and mimosine was precipitated at 4 °C overnight. The precipitate was recrystallized from 5 N NaOH (pH 9.0) and 6 N HCl (pH 4.5–5.0) and then allowed to stand at 4 °C to give pure mimosine. Mimosine was stored at −20 °C until further use. Mimosine was identified by LC-MS (*ESI*): *m*/*z* [M + H]^+^ 199.1 [13].

### 3.3. Synthesis of Mimosinol and Deuterated Mimosinol

Trifluoromethanesulfonic acid (187 μL, 2 mmol) was added to a 25-mL round-bottom flask containing 3.4 mL dichloromethane (CH_2_Cl_2_). After stirring at room temperature, tris(triethylsilyl)silane solution (618 μL, 2 mmol) was added dropwise, and the mixture was stirred at room temperature for 3 h until the solution became clear. Mimosine (0.4 g, 2 mmol) was placed in a round-bottom flask, to which imidazole (0.15 g, 2.2 mmol) and DMF: CH_2_Cl_2_ (3.4 mL, 1:1) were then added. The reaction flask was cooled to 0 °C, and tris(triethylsilyl)silyl triflate was added dropwise. After the addition was completed, the reaction was stirred at room temperature for 2 h. Mimosine ester was obtained from the filtrate by evaporation. A solution of sodium borohydride (0.28 g, 7.2 mmol) or sodium borodeuteride (0.3 g, 7.2 mmol) in 3 mL 50% ethanol was added to solution of mimosine ester in 3 mL of 50% ethanol. The resulting mixture was refluxed at room temperature for 5.5 h, and the solvent ethanol was evaporated *in vacuo*. The aqueous solution thus obtained was extracted with ethyl acetate (3 × 20 mL); the combined extracts were washed with saturated sodium chloride, dried over anhydrous sodium sulfate, and evaporated to give mimosinol (**1**) as a colorless crystal (352 mg, 95% yield) and deuterated mimosinol (d-mimosinol) (**2**) as a colorless liquid (274 mg, 73.5% yield). The NMR spectral data were in agreement with reported values for mimosinol and d-mimosinol described previously by Nguyen *et al*. [22]. LC-MS (*ESI*): *m*/*z* [M]^+^ 184.1, 186.1 for mimosinol and d-mimosinol, respectively.

### 3.4. General Procedure for Synthesis of Mimosinol *(**1a**–**c**)* Derivatives

The derivatives were prepared following procedure described by Tawata *et al*. [21] with modifications. A solution of mimosinol (1 mmol) and triethylamine (2 mmol) in 5 mL dioxane was cooled in an ice bath; thiophosphoryl chloride (1 mmol) in 5 mL dioxane was added dropwise, and the reaction mixture was stirred for 1 h. The resulting triethylammonium chloride was filtered off, and the filtrate was washed twice with dioxane. Sodium methoxide (1 mmol) in alcohol solution was added slowly to the filtrate. After stirring for 10 min, the solvents were evaporated, and the oily residue was dissolved in chloroform and washed twice with saturated sodium chloride solution. The organic layer was dried over anhydrous sodium sulfate and distilled to afford mimosinol derivatives (**1a**–**c**).

### 3.5. General Procedure for Synthesis of Deuterated Mimosinol *(**2a**–**c**)* Derivatives

The derivatives were synthesized following procedure described by Tawata *et al*. [21] with modifications. A solution of d-mimosinol (1 mmol) and triethylamine (2 mmol) in 10 mL dioxane was cooled in an ice bath, and thiophosphoryl chloride (1 mmol) in 10 mL dioxane was added dropwise. The reaction mixture was stirred for 10 min at a temperature <10 °C. After completion of the reaction, the triethylamine hydrochloride was filtered off, and the filtrate was obtained. A solution of alcohol (1.4 mmol), triethylamine (1 mmol) in 10 mL dioxane was added slowly to the filtrate. Approximately 100 min was required for displacement of a chlorine atom with a bulky alkyl group. The formed triethylamine hydrochloride was removed by filtration, and the filtrate was concentrated. Chloroform was added to the residue solution, and the chloroform solution was washed twice with saturated sodium chloride solution. The separated organic layer was dried over anhydrous sodium sulfate, and distilled to afford d-mimosinol derivatives (**2a**–**c**).

*3-Hydroxy-1-(((4S)-2-methoxy-2-sulfido-1,3,2-oxazaphospholidin-4-yl)methyl)pyridin-4(1H)-one* (**1a**): Pale yellow crystals, yield 34%. ^1^H-NMR (CDCl_3_, 400 MHz) δ: 1.29 (m, 2H, CH_2_CHNH), 3.29 (d, 3H, OCH_3_), 3.82 (m, 1H, CHNH), 4.06–4.13 (m, 2H, OCH_2_), 7.54 (s, 1H, CH), 8.20 (s, 1H, CH), 9.21 (s, 1H, CH). ^13^C-NMR (CDCl_3_) δ: 49.7 (s, CH_2_CHNH), 55.1 (d, OCH_3_), 58.3 (d, CHNH), 60.8 (d, CHNH), 63.3 (d, OCH_2_), 115.4 (s, CH), 120.4 (s, CH), 138.5 (s, CH), 166.3 (s, C), 181.8 (s, C). LC-MS (*ESI*): *m*/*z* [M]^+^ 276.1.

*1-(((4S)-2-Ethoxy-2-sulfido-1,3,2-oxazaphospholidin-4-yl)methyl)-3-hydroxypyridin-4(1H)-one* (**1b**): Pale yellow crystals, yield 39.6%. ^1^H-NMR (CDCl_3_, 400 MHz) δ: 1.30 (m, 2H, CH_2_CHNH), 3.29 (d, 3H, CH_2_CH_3_), 3.48 (s, 2H, OCH_2_), 3.78 (m, 1H, CHNH), 4.01–4.15 (m, 2H, OCH_2_), 7.54 (s, 1H, CH), 8.18 (s, 1H, CH), 9.22 (s, 1H, CH). ^13^C-NMR (CDCl_3_) δ: 16.0 (d, CH_3_), 50.6 (s, CH_2_CHNH), 56.3 (d, OCH2), 58.4 (d, CHNH), 59.4 (d, CHNH), 63.4 (d, OCH_2_), 116.4 (s, CH), 121.3 (s, CH), 138.1 (s, CH), 166.4 (s, C), 181.8 (s, C). LC-MS (*ESI*): *m*/*z* [M]^+^ 290.1.

*3-Hydroxy-1-(((4S)-2-propoxy-2-sulfido-1,3,2-oxazaphospholidin-4-yl)methyl)pyridin-4(1H)-one* (**1c**): Pale yellow crystals, yield 28.9%. ^1^H-NMR (CDCl_3_, 400 MHz) δ: 0.91 (m, 3H, CH_2_CH_3_), 1.29 (m, 2H, CH_2_CHNH), 1.70 (m, 2H, CH_2_CH_2_), 3.29 (dd, 2H, OCH_2_), 3.78 (m, 1H, CHNH), 4.05–4.12 (m, 2H, OCH_2_), 7.55 (s, 1H, CH), 8.17 (s, 1H, CH), 9.20 (s, 1H, CH). ^13^C-NMR (CDCl_3_) δ: 10.1 (d, CH_3_), 23.5 (m, CH_2_), 46.0 (s, CH_2_CHNH), 55.4 (d, OCH_2_), 56.8 (d, CHNH), 58.3 (d, CHNH), 63.3 (d, OCH_2_), 116.7 (s, CH), 121.2 (s, CH), 138.7 (s, CH), 166.3 (s, C), 181.1 (s, C). LC-MS (*ESI*): *m*/*z* [M]^+^ 304.5.

*3-Hydroxy-1-(((4S)-2-methoxy-2-sulfido-1,3,2-oxazaphospholidin-4-yl)methyl)pyridin-4(1H)-one* (**2a**): Pale yellow crystals, yield 15%. ^1^H-NMR (CDCl_3_, 400 MHz) δ: 1.30 (m, 2H, CH_2_CHNH), 3.31 (d, 3H, OCH_3_), 3.83 (m, 1H, CHNH), 7.54 (s, 1H, CH), 8.19 (s, 1H, CH), 9.22 (s, 1H, CH). ^13^C-NMR (CDCl_3_) δ: 49.4 (s, CH_2_CHNH), 54.9 (d, OCH_3_), 58.0 (d, CHNH), 60.2 (d, CHNH), 63.1 (d, OCH_2_), 115.7 (s, CH), 120.2 (s, CH), 137.7 (s, CH), 166.0 (s, C), 180.9 (s, C). LC-MS (*ESI*): *m*/*z* [M]^+^ 278.1.

*1-(((4S)-2-ethoxy-2-sulfido-1,3,2-oxazaphospholidin-4-yl)methyl)-3-hydroxypyridin-4(1H)-one* (**2b**): Pale yellow crystals, yield 12%. ^1^H-NMR (CDCl_3_, 400 MHz) δ: 1.31 (m, 2H, CH_2_CHNH), 3.32 (d, 3H, CH_2_CH_3_), 3.50 (m, 2H, OCH_2_), 3.77 (m, 1H, CHNH), 7.54 (s, 1H, CH), 8.18 (s, 1H, CH), 9.23 (s, 1H, CH). ^13^C-NMR (CDCl_3_) δ: 15.8 (d, CH_3_), 50.3 (s, CH_2_CHNH), 56.1 (d, OCH2), 58.4 (d, CHNH), 59.2 (d, CHNH), 63.2 (d, OCH_2_), 116.1 (s, CH), 121.0 (s, CH), 137.9 (s, CH), 166.1 (s, C), 181.5 (s, C). LC-MS (*ESI*): *m*/*z* [M]^+^ 292.4.

*3-Hydroxy-1-(((4S)-2-propoxy-2-sulfido-1,3,2-oxazaphospholidin-4-yl)methyl)pyridin-4(1H)-one* (**2c**): Pale yellow crystals, yield 10%. ^1^H NMR (CDCl_3_, 400 MHz) δ: 0.90 (m, 3H, CH_2_CH_3_), 1.30 (m, 2H, CH_2_CHNH), 1.68 (m, 2H, CH_2_CH_2_), 3.28 (dd, 2H, OCH_2_), 3.77 (m, 1H, CHNH), 7.56 (s, 1H, CH), 8.20 (s, 1H, CH), 9.19 (s, 1H, CH). ^13^C-NMR (CDCl_3_) δ: 10.0 (d, CH_3_), 23.3 (m, CH_2_), 45.8 (s, CH_2_CHNH), 55.2 (d, OCH_2_), 56.8 (d, CHNH), 58.1 (d, CHNH), 63.1 (d, OCH_2_), 117.7 (s, CH), 121.2 (s, CH), 138.2 (s, CH), 165.9 (s, C), 181.4 (s, C). LC-MS (*ESI*): *m*/*z* [M]^+^ 306.5.

### 3.6. Topical Assay against Termites

An acute toxicity bioassay was performed by topical application to worker termites. A series of seven different doses (0.25, 0.5, 1.5, 2.5, 7.5, 12.5, and 25 μg/termite) was prepared in ethanol solution for each tested sample. Aliquots (0.5 μL) of diluted samples were applied topically to the abdomens of worker termites. Controls were treated with 0.5 μL ethanol only. Termites were transferred into petri dishes (4.2-cm diameter) lined with filter paper and kept in an incubator at 23–25 °C. A few drops of distilled water were supplied daily to the bottom edge of each dish to maintain moisture during the experiment. Four replicates of 20 termites each were used per treatment. Fenitrothion and rotenone were used as positive controls. Mortality of insects was evaluated after 48 h treatment. Insects were considered dead when they became immobilized and did not respond to external stimuli. LD_50_ values were calculated by probit analysis using Graphpad Prism 6.01 (GraphPad, La Jolla, CA, USA).

### 3.7. No-Choice Assay against Termites

The no-choice bioassay method as described by Tawata *et al*. [23] was used to evaluate insecticidal activity. The tested compounds were dissolved in ethanol and acetone to three concentrations (50, 100, 250 μg/mL), and applied to filter paper that was placed in petri dishes (8.5-cm diameter). Filter paper treated with ethanol and acetone was used as the control. After removing the solvent from treated filter paper by air drying at ambient temperature for 24 h, 20 termites were placed onto each paper. The dishes were covered and placed in incubator at ±23 °C. A few drops of distilled water were supplied daily to the bottom edge of each petri dish to maintain moisture during the experiment. The experiment was performed using four replicates of each treatment and was repeated two times. Mortality was evaluated after seven days treatment. Insects were considered dead when they became immobilized and did not respond to external stimuli. Insect mortality rate was calculated as a percentage in comparison with the control group. Rotenone and fenitrothion were used as positive controls.

### 3.8. Nematicidal Activity Assay against Caenorhabditis Elegans

The nematicidal activity assay was conducted according to previously described procedures with minor modifications [24,25]. The nematode *Caenorhabditis elegans* was cultivated on nematode growth medium (NGM) [3 g NaCl, 15 g agar, 2.5 g polypeptone, 136.1 g KH2OP4, 17.9 g KOH, 1 mL 1 M MgSO4, 1 mL 1 M CaCl2, 1 mL cholesterol (5 mg/mL), and 500 μL ampicillin (100 μg/mL)] plate covered with *Escherichia coli* strain OP50. The ampicillin-resistant OP50 was used as a feed source to prevent cross-contamination of the nematode culture. The NGM plates contained high densities of worms after 4 days incubation at 20 °C. For preparation of synchronous worm culture, worms were rinsed from the agar plate with S-basal buffer (5.85 g NaCl, 1 g K_2_HPO_4_, 6 g KH_2_PO_4_, and 1 mL cholesterol [5 mg/mL ethanol]/L), washed twice with S-basal buffer, and added to household bleach/10 N NaOH solution. The supernatant was shaken for 10–15 min, washed three times by centrifugation for 1 min at 3000 rpm, and then resuspended in S-basal buffer. One hundred microliters of worm/OP50 (1.2 × 10^9^ bacteria/mL) solution was transferred into each well of a 96-well plate, and 20 μL sample stock solution was added to give final concentrations 25, 50, 100, 250, and 500 μM. The plate was shaken thoroughly for 2–3 min and incubated at 20 °C. Water was used as a negative control. The experiment was performed using four replicates of each treatment and was repeated two times. Dead and active nematodes were recorded after 48 h treatment. Nematode mortality rate was calculated as a percentage in comparison with the control group.

### 3.9. Assay for Acetylcholinesterase (AChE) Inhibition

Whole bodies of termites (20 mg) were homogenized in 1 mL of 0.1 M phosphate buffer (pH 8.0), and the homogenate was centrifuged at 12,000 rpm for 20 min. The supernatant was used as the enzyme source. All procedural steps for preparing the crude enzyme were performed at 4 °C [26]. The AChE activity was measured using the modified Ellman’s method [27]. Twenty-five microliters of sample was transferred into each well of a 96-well microplate containing 125 μL 0.1 M phosphate buffer (pH 8.0), and 30 μL of the enzyme solution was added to each well. The mixture was incubated for 10 min at 25 °C, after which 50 μL of 5,5′-dithiobis-(2-nitrobenzoic acid) (final concentration 0.4 mM) and 25 μL of acetylthiocholine iodide (final concentration 1 mM) were added. The control was treated by adding 25 μL water. Enzyme activity was measured for 20 min at 412 nm. The AChE inhibition assay was performed using four replicates of each treatment and was repeated two times. The inhibition rate of AChE activity was calculated as a percentage as follows:

Inhibition (%) = (A_o_ − A_E_)/A_o_ × 100 (1)

where A_o_ is the absorbance of the control, and A_E_ is the absorbance of the tested sample.

### 3.10. Tyrosinase Inhibition Assay

A microplate assay for tyrosinase inhibitory activity was performed following a previously described procedure [28]. Samples (20 μL) with various concentrations were transferred into each well of a 96-well plate; 120 μL of 20 mM sodium phosphate buffer (pH 6.8) and 20 μL of 500 U/mL mushroom tyrosinase enzyme dissolved in buffer were then added to each well. The mixture was incubated at 25 °C for 15 min, after which 20 μL of 0.85 mM l-tyrosine solution was added. Absorbance was recorded at 470 nm using a microplate reader (Benchmark plus; Biorad, Hertfordshire, UK). Mimosine and kojic acid were used as a positive controls. The percentage of inhibition was calculated as follows:

Inhibition (%) = [(C_E_ − C_o_) − (S_E_ − S_o_)]/(C_E_ − C_o_) × 100 (2)

where C_E_ is the absorbance of the control with enzyme, C_o_ is the absorbance of the control without enzyme, S_E_ is the absorbance of the tested sample with enzyme, and S_o_ is the absorbance of the tested sample without enzyme.

### 3.11. Data Analysis

Statistical analyses were performed using statistical analysis system (SAS) software, version 9.1.3 (SAS Institute Inc., Cary, NC, USA). Significance was assessed by one-way ANOVA analysis, and means were separated using Duncan’s test at *p* ≤ 0.01. All calculations were conducted in Microsoft Excel 2003. The IC_50_ values were determined graphically as the concentration of each compound that showed 50% inhibitory activity.

## 4. Conclusions

In conclusions, this report provides the first evidence for insecticidal and nematicidal activities of novel mimosine derivatives. Mimosinol and d-mimosinol could be potential bioinsecticides which had strong insecticidal activity by inhibiting tyrosinase. Among phosphoramidothionate derivatives prepared, compounds **1a** and **1b** showed promise as having both of these activities. Our findings also indicate that mimosine was a good nematicidal compound. Appropriate length of the alkyl chain and the functional group at the C_5_-position of phosphoramidothionates derived from mimosinol and d-mimosinol are important for conferring insecticidal and nematicidal activities. These results introduce mimosine as previously unexplored scaffold of insecticide and nematicide.
